# Influence of Electrical and Ionic Conductivities of Organic Electronic Ion Pump on Acetylcholine Exchange Performance

**DOI:** 10.3390/ma10060586

**Published:** 2017-05-26

**Authors:** Nazrin Abdullayeva, Mehmet Sankir

**Affiliations:** Department of Materials Science and Nanotechnology Engineering, Tobb University of Economics and Technology, Sogutozu C. No. 43 Sogutozu 06560 Ankara, Turkey; nabdullayeva@etu.edu.tr

**Keywords:** ion pump, acetylcholine, electrically conductive polymers, PEDOT:PSS, ionomers

## Abstract

By using an easy and effective method of depositing conjugated polymers (PEDOT:PSS) on flexible substrates, a new design for organic bioelectronic devices has been developed. The purpose was to build up a system that mimics the motion of neurotransmitters in the synaptic cleft by obtaining an electrical to chemical signal transport. Fourier transform infrared (FTIR) spectroscopy and Raman measurements have demonstrated that electrochemical overoxidation region which separates the pristine PEDOT:PSS electrodes and allows ionic conduction has been achieved successfully. The influence of both electrical and ionic conductivities on organic electronic ion pump (OEIP) performances has been studied. The ultimate goal was to achieve the highest equilibrium current density at the lowest applied voltage via enhancing the electrical conductivity of PEDOT:PSS and ionic conductivity of electrochemically overoxidized region. The highest equilibrium current density, which corresponds to 4.81 × 10^17^ number of ions of acetylcholine was about 41 μA cm^−2^ observed for the OEIP with the electrical conductivities of 54 S cm^−1^. This was a threshold electrical conductivity beyond which the OEIP performances were not changed much. Once Nafion™ has been applied for enhancing the ionic conductivity, the equilibrium current density increased about ten times and reached up to 408 μA cm^−2^. Therefore, it has been demonstrated that the OEIP performance mainly scales with the ionic conductivity. A straightforward method of producing organic bioelectronics is proposed here may provide a clue for their effortless mass production in the near future.

## 1. Introduction

Recent advancements in the electrochemical applications of conjugated polymers have increased the demand for their utilization in several progressive fields such as biosensors [1,2], actuators [3,4], transistors, and flexible electronics [5]. A special interest was addressed to poly(3,4-ethylenedioxythiophene) polystyrene sulfonate (PEDOT:PSS), a transparent, conductive polymer, which is suitable for utilization in flexible electronic devices for their high ductility and fair conductivity. With the development of organic electronic devices for the last few years, several methods have been proposed in order to properly incorporate PEDOT:PSS into these systems. The main reason of PEDOT:PSS drawing so much attention is because it combines both ionic and electrical conductivity where the PEDOT part provides the electrical conductivity of the conjugated polymer, while the PSS offers ionic transport. Several studies in the area of organic electronic devices have been conducted [2,6,7]. Some of them are mainly based on the construction of the organic electronic ion pump (OEIP), a four-electrode delivery system designed to transfer ions from one subsystem to another via electrophoretic motion, thus mimicking the synaptic transfer in neural cells [6,7,8]. The system consists of selectively-deposited PEDOT:PSS electrodes on poly(ethylene terephthalate) (PET) substrates with a tiny overoxidized region which serves as an electrically insulating, but ionically conductive, part. The study is focused on translation of electronic signals into ion fluxes and obtaining an intracellular communication, thus mimicking the work of a neural cell. In similar studies PEDOT:PSS on flexible substrates are utilized with several methods. Processes like screen-printing, resist mask printing, UV lithography, and photolithography have been among the methods developed for PEDOT:PSS deposition on several substrates [5,9,10,11]. However, even in these highly-advanced methods several drawbacks, such as tuning the photoresist thickness, time of exposition and development, and reproducibility, constantly occur. In some cases multiple steps of patterning processes hinder the larges-scale production of these devices. 

Although this field of study is quite advanced, there are still some restrictions related to the performance of organic electronic devices for their low release rates, high voltage demands, and limitations in design parameters. Therefore, the preparation procedure of OEIP from PEDOT:PSS for electronic control of ionic groups usually exchanged in neuronal cells is further improved in our laboratory. In this study a straightforward, but cost-effective and time-saving, method called line patterning is used to selectively deposit PEDOT:PSS conjugated polymer on PET substrate at desired shapes required for better ion pump performances. Previous studies done in the area of electronic applications of poly(3,4-ethylenedioxythiophene) polystyrene sulfonate (PEDOT:PSS) have investigated the effects of several additives on its conductivity values. The main purpose of those studies was to tune electrical conductivity of PEDOT:PSS by using several organic solvents and salts in order to obtain maximum conductivity. The electrical conductivities of PEDOT:PSS used in the area of transistors and other electronic devices has been reported in the range of 5–300 S cm^−1^ [12,13,14]. For the organic electronic ion pump (OEIP) structure, however, these conductivity values have never been reported. In these studies it was indicated that the utilized PEDOT:PSS solution was of high conductivity grade [6,7,8]. This is important to point out because in our study PEDOT:PSS granules were dissolved in water to obtain PEDOT:PSS solution by our own method thus giving out a solution with different conductivity features. Consequently, the effect of additives for conductivity enhancement affected the solution differently. As we already know, PEDOT:PSS is both an electrically- and ionically-conductive polymer. Although there are many studies based on the electrical conductivity of PEDOT:PSS and its enhancement [12,13,14], to the best of our knowledge there is no study for measuring the ionic conductivity of PEDOT:PSS where its electrical conductivity is hindered by chemical/electrochemical overoxidation. In our study, in addition to tailoring the conductivity of PEDOT:PSS, an ionomer has been investigated as well. Ionomers are types of polymers containing partially-charged ionic groups in their structures. These materials are best known for their perfect ionic transport properties. Among these materials Nafion^TM^, is the most frequently used ionic conductor that has been studied extensively specifically for fuel cells and flow batteries [15,16]. Nafion^TM^ is known to be an ionically conductive, but electrically insulating, material. Tuning the ionic conductivity of the overoxidized region of OEIP was performed by using Nafion^TM^. As far as we know from the literature, OEIP systems have been studied previously [6,7,8], but have never been investigated in the depth necessary to define the several critical parameters, such as ionic and electrical conductivities, and the influences of them on the OEIP performance. Moreover, there was no specific study focusing on the ionic conductivity properties of PEDOT:PSS. In conclusion, this study is reporting the tuned ionic and electrical conductivity of the structure strongly affecting the performance of OEIP.

## 2. Experimental 

### 2.1. Materials

As the substrate of OEIP, polyethylene terephthalate (PET) was selected as the most suitable one for its being flexible, biocompatible, and electrically-insulating. PEDOT:PSS, in granule form, and ethylene glycol (EG) and hydrochloric acid were purchased from Sigma Aldrich (Schnelldorf, Germany). Glycerol (Sigma Aldrich, Schnelldorf, Germany) was selected as an additive to the PEDOT:PSS solution for promoting better adhesion on the PET substrate. Potassium chloride (KCl), calcium chloride (CaCl_2_), and acetylcholine chloride (AChCl) necessary for the potentiostatic experiments were purchased from Sigma Aldrich, as well. For the line-patterning material, standard printer toner was used. It was selected as the most appropriate material for tuning the surface wettability providing a hydrophobic region for holding off the PEDOT:PSS solution. Isopropyl alcohol (IPA) and acetone were purchased from Sigma Aldrich and used as received. 

### 2.2. Line Patterning and PEDOT:PSS Deposition

PET picked out as a good flexible substrate eligible for utilization in biological systems, was pre-treated with acetone and isopropanol (IPA) before being patterned. Before contouring the desired shapes on the substrate, it passed through one more step called air plasma treatment. Air plasma treatment or, in other words, corona treatment, is a technique used for surface modification by using corona discharge plasma. Under the application of high voltage plasma is generated through the tip of the device changing the surface energy of the substrate, providing an enhanced bonding during deposition. The treated PET substrate, ready to use, is patterned with the printer toner forming the hydrophobic regions and allowing a determinate space for the ink to be injected.

PEDOT:PSS solution was prepared by dissolving granules in water (0.25 % *w*/*w*) and by adding glycerol (5% *v*/*v*) in order to enhance the surface adhesion of solution. The deposition of the PEDOT:PSS solution on the substrate is done by a standard drop-cast method on an area of 1.0 cm^2^. Due to the presence of the hydrophobic phase on the substrate, the PEDOT:PSS solution diffuses to the available regions only taking the desired shape of the pattern. The solution is dried on a hot plate for 30 min at 50 °C and 2 h at 100 °C. After the overall deposition is actualized, the electrochemical overoxidation step proceeds.

### 2.3. PEDOT:PSS Electrochemical Overoxidation

**Figure 1:**
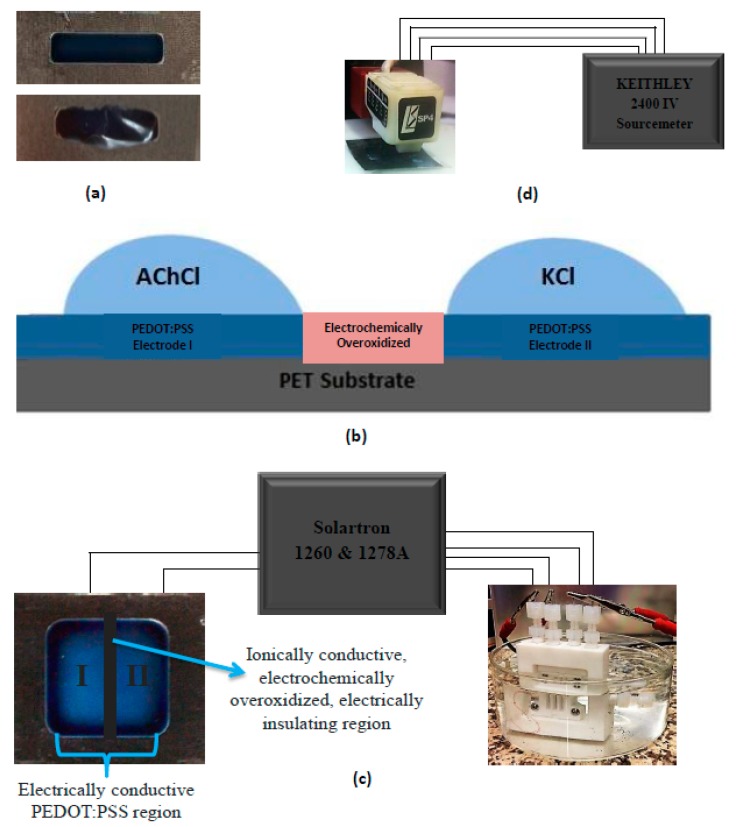
(**a**) Difference in PEDOT:PSS deposition between corona-treated and non-treated PET substrate; (**b**) cross-sectional image of I and II electrodes on the PET substrate with a liquid electrolyte of AChCl and KCl; and (**c**) configuration of OEIP and conductivity cell connected to Sourcemeter Solartron 1260 and 1278 A. Opposing PEDOT:PSS electrodes (I and II) serve as the base for AChCl and KCl solutions. The middle region is electrochemically overoxidized allowing only ionic transport; and (**d**) a four-point probe resistivity measurement device connected to Keithley 2400 IV sourcemeter.

The OEIP system is separated into three main regions. Two of them, labeled as I and II, are pristine PEDOT:PSS layers which serve as electrodes. The other region is an electrochemically-overoxidized region which is located in between region I and II. Under the applied voltage among electrodes I and II, acetylcholine (ACh^+^) ions are driven from region I to region II through the electrochemically-overoxidized zone (Figure 1). Electrochemical overoxidation of PEDOT:PSS is an essential step in OEIP design. This step permanently cuts off the electrical conductivity of the PEDOT phase making that part of system conductive to ions only. The region serves as a good ionic conductor leading to an easy transport of positively-charged ions. Under the effect of voltage, ions located on one side of electrochemically-overoxidized region are transported through it to the opposite side. PEDOT:PSS overoxidation is done by electrochemical method, which is assumed to be safer, faster, and easily controlled in comparison to chemical overoxidation achieved by sodium hypochlorite [14,17]. Samples were electrochemically overoxidized in a two-electrode cell with a stainless steel wire as a counter electrode where approximately 15 V was applied between the KCl electrolyte solution and the counter electrode. Since the overoxidation is done in a determinate region, the electrolyte solution is dropped directly onto that part of the PEDOT:PSS, setting the boundaries with insulating polymers. The total overoxidized area is about 32 mm^2^.

### 2.4. Potentiostatic Measurements and Cycle Tests

After constructing the OEIP properly, electrolyte solutions (AChCl and CaCl_2_ both 0.1 M) were dropped on pre-determined opposing regions. Under the effect of constant voltage, K^+^ ions from one electrode pass through overoxidized region to the opposite electrode. As sourcemeter Solartron 1260 and 1278 A was used to apply a constant voltage. In order to test the performance of OEIP at different conditions, side I and II were filled with AChCl (0.1 M) and KCl (0.1 M) solutions, respectively, and several potentials, such as 1, 2, 5, and 10 V, were applied for electrodes I and II and current vs. time graphs were analyzed. The number of charge values was calculated from the current versus time graphs. This was performed by a well-known “Cumulative trapezoidal numerical integration” (Cumtrapz) function in MATLAB 2013 computer aided software (R2013b version 8.2, MathWorks^®^, Natick, MA, USA). Then, by using Equation (1), the number of transported ions was calculated:

(1) $$\mathrm{Number}\;\mathrm{of}\;\mathrm{transported}\;\mathrm{ions}=\;\frac{1\;\mathrm{mole}\;\times \mathrm{Charge}\;C}{96500\;C}\;\times 6.02\times 10^{23},$$

The cyclic performance tests have been achieved for the device with the best OEIP performance include measuring the equilibrium current densities at 1.0 V with each 5 min interval in which AChCl (0.1 M) and KCl (0.1 M) solutions on the electrodes have been refreshed after each voltage cycle (1.0 V).

### 2.5. Fourier Transform Infrared (FTIR) and Raman Spectroscopy 

It was assumed that the electrochemical overoxidation of PEDOT:PSS as a result leads to cutting off the electrical conductivity of overall conjugated polymer would end up in significant changes in its chemical structure. This was analyzed by the FTIR measurements (Perkin Elmer Spectrum 100, Perkin Elmer, Shelton, CT, USA). Another characterization technique for the electrochemical overoxidized region of PEDOT:PSS was suggested by the Raman spectroscopy (Renishaw, India Reflex, New Mills, UK) which was expected to show the main peak shifts prior to and after electrochemical overoxidation steps. Raman measurements were performed with a 532 nm Ar-ion laser. 

### 2.6. Conductivity Enhancement

Once the standard potensiostatic tests have been performed, several methods were developed in order to strengthen the electrical and proton conductivity of PEDOT:PSS. As the first method, EG treatment was chosen. Drop-wise-added EG on electrodes was maintained at different time intervals of 2, 6, 12, and 24 h, and samples were annealed in a vacuum oven at 100 °C for 30 min. As an additional technique for conductivity enhancement, acid treatment was selected. The pH of the PEDOT:PSS solution was modified with hydrochloric acid. Electrical conductivities of OEIPs after the abovementioned treatments were measured by using a four-point probe resistivity measurement device equipped with a Keithley 2400 IV sourcemeter. The working principle of the four-point probe is based on a fixed applied current between two outer probes, and voltage that is measured between two other inner probes. After applying a sweep voltage between −1 and +1 V current vs. voltage data sets were obtained and, as a result, resistivity values could be evaluated.

On the other hand, improving ionic conductivity was also aimed in our study, thus achieving better acetylcholine ion transfer through the electrochemically-overoxidized region. For this purpose, the electrochemically-overoxidized region was covered with 5% Nafion™ aqueous solution and dried under an IR lamp for 30 min. Proton conductivities at 25 °C for the fully-hydrated samples were measured with a conductivity cell via Solartron 1260 and 1278A.

## 3. Results and Discussion

**Figure 2:**
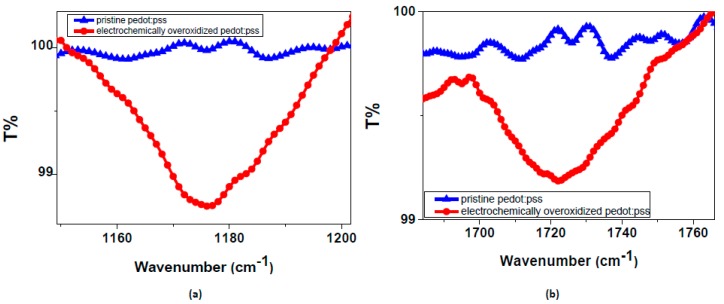
FTIR spectra of pure and overoxidized PEDOT:PSS. The most significant peak shifts in (**a**) represents S=O stretching at 1175 cm^−1^ and (**b**) C=O stretching at 1720 cm^−1^.

The deposition of PEDOT:PSS on flexible PET substrate in pre-determined shapes was done by a line-patterning method, followed by drop-casting [5,9]. An important parameter playing a crucial role at this step is corona treatment that provides an enhanced adhering of conjugated polymer on the substrate by modifying the surface energy. The images of corona-treated versus corona-non-treated PEDOT:PSS deposited on PET substrate are given in Figure 1a. The difference in adhesion is clearly seen between two images. As a result, defect-free well-adhered samples were successively achieved after surface corona treatment. As seen in Figure 1b, the OEIP includes an overoxidized region between electrodes I and II. The main role of this region is to electrically insulate the gap between electrode I and II and transport ions from electrode I to II. The overoxidized region should only conduct ions, not electrons, therefore, a potential can be applied between electrode I and II. Overoxidation in this study was successfully achieved electrochemically. Spectra obtained from FTIR measurements were analyzed and significant differences were observed due to the electrochemical overoxidation. It is obvious from Figure 2 that prior to overoxidation there is no peak on the pure PEDOT:PSS spectra, while a prominent peak occurs after overoxidation. The peak occurring close to 1720 cm^−1^ corresponds to C=O stretching, while double-head peaks in the 1175 cm^−1^ wavenumber range are attributed to the sulfonate group (S=O) stretching. These peaks in the FTIR spectra explain the lowering of the electrical conductivities of PEDOT:PSS to about 0.06 S cm^−1^. According to previous studies done in this area, this change corresponds to the formation of carbonyl groups on thiophene rings that cut off the conjugation in the chains [18,19,20]. The remaining chemical structure then becomes electrically insulating, but ionically conductive due to the polystyrene sulfonate groups (PSS). In conclusion, the electrochemical overoxidation has been successfully achieved.

**Figure 3:**
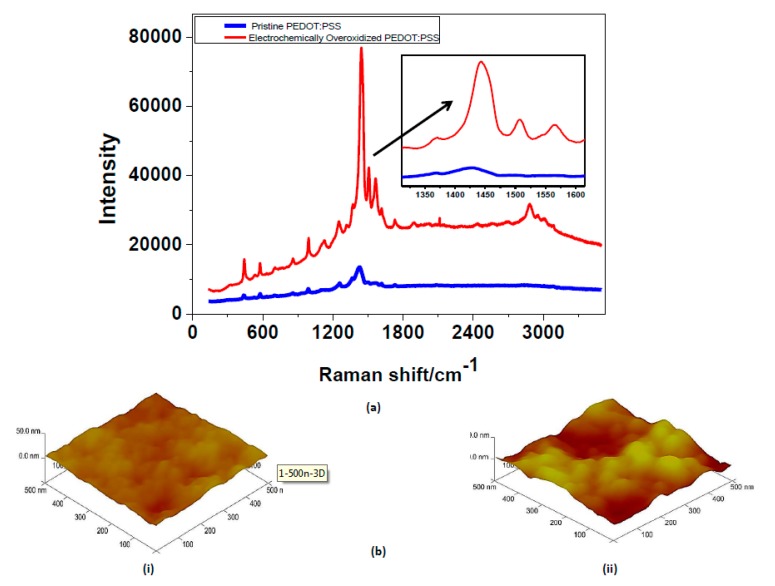
(**a**) Raman spectra for pristine and overoxidized PEDOT:PSS. Peaks at 1430 cm^−1^ correspond to the characteristic C=C stretching of the thiophene ring, which shifts and intensifies after the overoxidation step; (**b**) AFM images of (i) pristine and (ii) electrochemically-overoxidized PEDOT:PSS. A severe increase in surface roughness is clearly seen.

The Raman spectra of pristine and electrochemically overoxidized PEDOT:PSS are given in Figure 3. There is a visible peak shift at wavenumber of approximately 1430 cm^−1^, which corresponds to the symmetric C=C stretching of the thiophene ring [21,22]. After the electrochemical overoxidation step, this peak shifts slightly towards 1450 cm^−1^, proving the oxidized state of the PEDOT:PSS. In the previous studies [21,22], this type of change in wavenumber and peak intensity increase was correlated to the transformation of the resonance structure of the thiophene backbone. Enhanced peaks at 1500 and 1570 cm^−1^ are also reported as asymmetric stretching vibrations of the thiophene ring of PEDOT:PSS in the middle and at the end of the chains. A significant change in surface topography of PEDOT:PSS was expected after the electrochemical overoxidation. AFM imaging was selected as the most proper method for determining the surface roughness values. The root mean square (RMS) surface roughness, *R*_q_, of pristine PEDOT:PSS at 500 nm scaling in AFM were reported in Table 1. Pristine PEDOT:PSS films appear to be highly homogeneous with extremely low roughness values. Due to the electrochemical overoxidation, the surface resulted in high roughness overall. Together with the AFM results, the change in the surface morphology was proven by performing contact angle measurements. A pristine and overoxidized PEDOT:PSS by using a simple contact angle method was tested and, eventually, the change in surface energy was interpreted. According to the contact angle measurements, hydrophilicity of the surface increases significantly after the electrochemical overoxidation step. This may correspond to the decrease in surface energy after electrochemically overoxidizing PEDOT:PSS. Therefore, the overoxidized region becomes more hydrophilic with the lower surface energy enhancing the aqueous ionic transport from electrode I to II. As a result, electrochemical overoxidation was effectively accomplished resulting in changing the surface energy and morphology.

**Figure 4:**
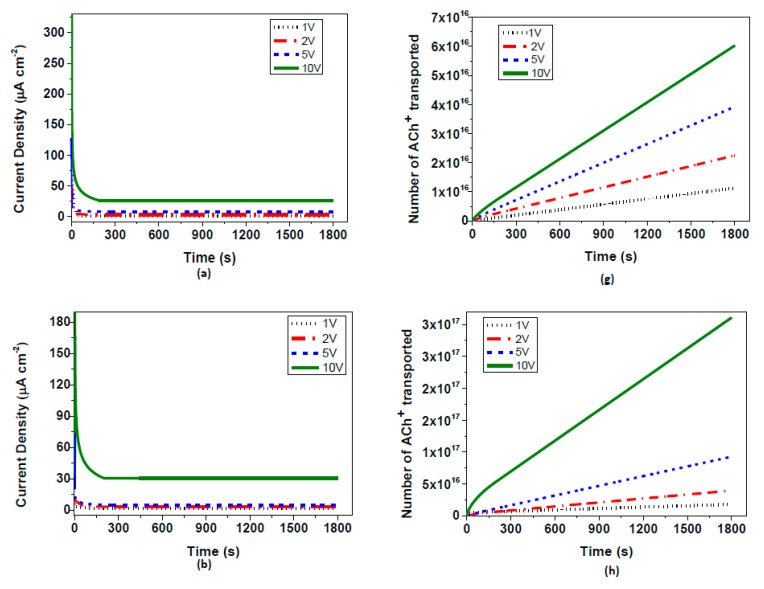

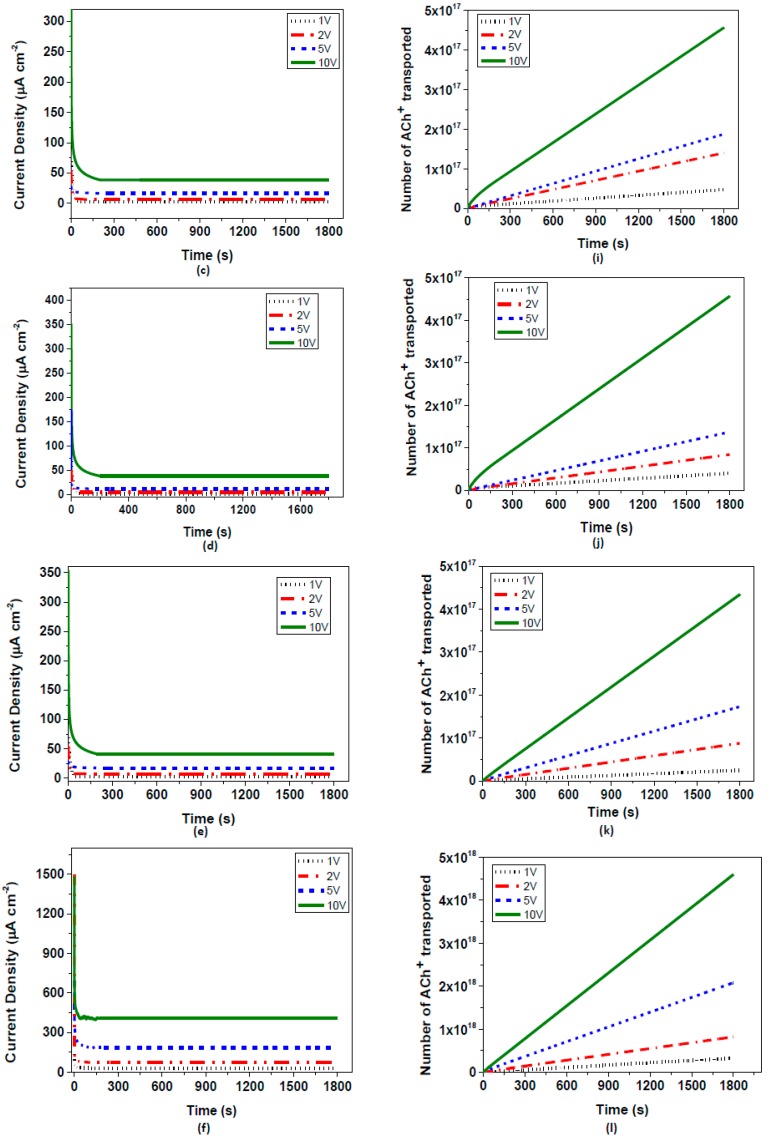
Current vs. time measurements at different applied voltages between two electrodes containing AChCl and KCl solutions oppositely for (**a**) pristine; (**b**) 2 h EG-treated; (**c**) 6 h EG-treated; (**d**) pH = 1.5, HCl acid-treated; (**e**) pH = 0.9, HCl acid-treated; (**f**) electrochemically-overoxidized region Nafion™-treated PEDOT:PSS electrodes; and the number of transported ACh+ ions at different applied voltages for (**g**) pristine; (**h**) 2 h EG-treated; (**i**) 6 h EG treated; (**j**) pH = 1.5, HCl acid-treated; (**k**) pH = 0.9, HCl acid-treated; and (**l**) electrochemically-overoxidized region Nafion™-treated PEDOT:PSS electrodes.

**Figure 5:**
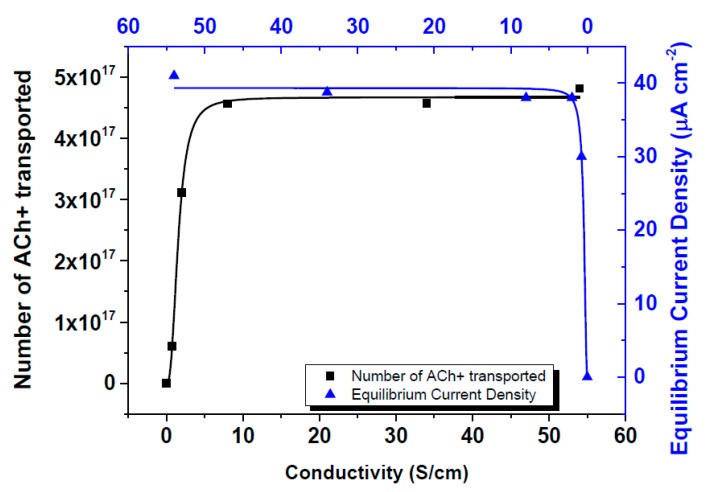
Influence of electrical conductivity of electrodes on the equilibrium current density and number of acetylcholine ions transported.

In order to study the influence of the electrical conductivity on the ion pump performances, the electrical conductivities of the PEDOT:PSS electrodes have been tailored. In previous studies performed in the area of the conductivity enhancement of PEDOT:PSS, several additives and/or thermal annealing processes have been well investigated [12,13,14,23,24,25]. Among them, ethylene glycol (EG) in this study was utilized for the conductivity enhancement experiments. By exposing only the electrodes I and II to EG solution for 5 min, the conductivities of the electrodes were expected to increase, while the electrochemically-overoxidized part was left unaffected. The explanation of increasing the electrical conductivity of PEDOT:PSS electrodes lies under the fact that EG abolishes the PSS phase that accounts for ionic transfer, thus leaving the polymer film with the remaining electrical conduction [5,14]. This way, ions could be driven faster towards the overoxidized region, speeding up the overall transfer. Electrical conductivity values of PEDOT:PSS electrodes are reported in Table 2. The effect of EG treatment time on the conductivities of I and II electrodes was also tested. For this purpose, PEDOT:PSS electrodes were exposed to EG for 2, 6, 12, and 24 h for tailoring the electrical conductivities of them. Although there was a very small change after 2 h of EG exposure, a significant increase in conductivity value from 0.38 S/cm to 8 S/cm was noticed under 6 h exposure. However, the conductivity increase stabilized after 6 h of treatment. Another additional method for conductivity enhancement of PEDOT:PSS was the pH drop with the hydrochloric acid treatment. Whilst the pristine PEDOT:PSS solution has a pH value of nearly 4.5, by decreasing this value to 1.5, and lower to 0.9, a significant increase is also seen in the conductivity values which can be summarized in Table 2. As previously mentioned, the performance of OEIP was tested at various applied potentials between the two electrodes. Therefore, the system was tested at 1 V, 2 V, 5 V, and 10 V, and current vs. time graphs were obtained (Figure 4). The application of OEIP is aimed for use in bioelectrochemical devices. For this reason its ability to transfer macromolecules, such as neurotransmitters, gives us a better understanding of the performance of OEIP later in vivo experiments. Acetylcholine transport has been monitored by the equilibrium current density, indicating the rate of drift of the acetylcholine ions from electrode I to electrode II at several potentials. At the highest potential of 10 V, nearly 25 µA cm^−2^ equilibrium current density has been observed. As previously mentioned, the influence of EG on PEDOT:PSS is towards enhancing its electrical conductivity. This provides us with a better transfer of acetylcholine ions through I and II electrodes, thus accumulating larger amounts of ions to be transferred through the electrochemically-overoxidized region. Therefore, we have observed that the equilibrium current density increased from 25 to 38 µA cm^−2^ when the electrical conductivities varied from 0.38 to 8 S/cm. An additional approach to the conductivity increase of PEDOT:PSS electrodes was acid treatment. By using hydrochloric acid, the pH value of the pristine PEDOT:PSS solution was dropped to 1.5 and then 0.9, which significantly increased the conductivity to 34 and 54 S/cm, respectively. Corresponding equilibrium current densities were then observed at about 39 and 41 µA cm^−2^, respectively (Figure 5). In comparison to previous studies investigating the OEIP structure [6,7,8], our equilibrium current values have vastly increased as a result of the enhancement of both electrical and ionic conductivities. Taking 10 V as the basis of the applied potential, previous studies have obtained a maximum of 20–30 µA current values, while in our system almost reached 41 µA via electrical conductivity treatment. Additionally, after Nafion™-treated overoxidized region of the OEIP structure, these equilibrium current values reached 408 µA showing that the performance of the system increased 10 times by ionic conductivity enhancement. As a conclusion, it has been demonstrated that the equilibrium current densities of OEIP reach a threshold value when the electrical conductivities of the electrodes reach 8 Scm^−1^, beyond which the equilibrium current densities remained almost constant at nearly 40 µA cm^−2^.

**Figure 6:**
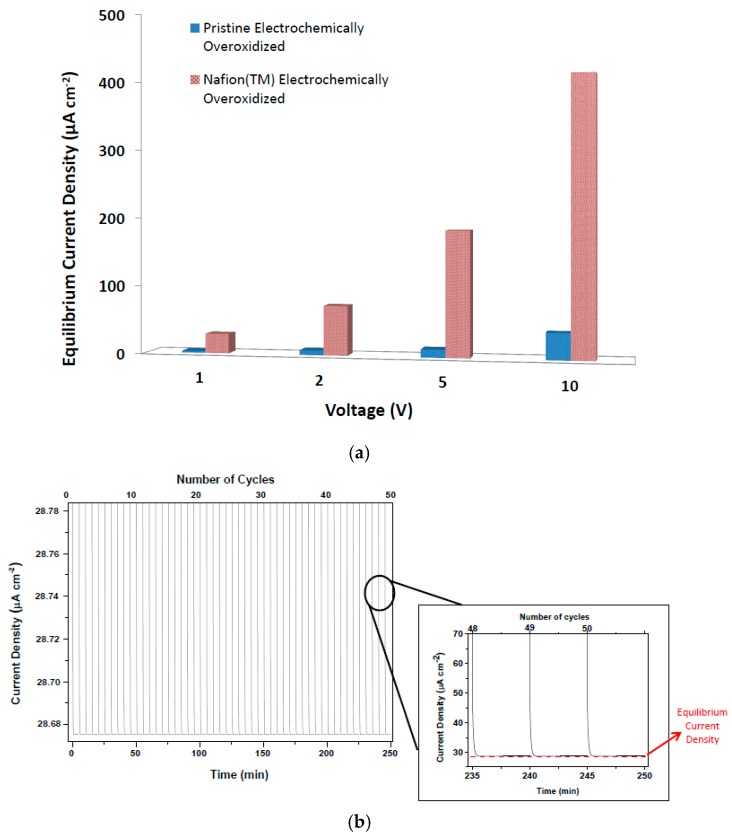
(**a**) Equilibrium current density values for OEIP with pristine and Nafion™-treated overoxidized regions; and (**b**) cyclic tests for Nafion™-treated PEDOT:PSS over 50 cycles.

The final treatment of OEIP was applied to the PEDOT:PSS electrodes with the highest equilibrium current density performance. This treatment method is based on enhancing the ionic transport of oxidized region of OEIP structure by adding Nafion™ layers on it. As can been seen in Figure 6, the equilibrium current density at all four voltage values have increased significantly, leading us to the conclusion that the effect of ionic conductivity is larger than assumed. The equilibrium electrode density of OEIP was increased nearly 10 times and reached up to 408 µA cm^−2^, indicating very fast acetylcholine transport from electrode I to II. The effect of Nafion™ as an ionic conductor to the OEIP structure can be interpreted as enhancement in proton conductivity of the electrochemically-overoxidized region. As seen in Table 3, the proton conductivities of the electrochemically-overoxidized region are significantly increased from 0.0035 to 0.13 S/cm. Therefore, it has been stated that the influence of ionic conductivity of the electrochemically-overoxidized region on OEIP performance is much greater than that of electrical conductivities of the electrodes. 

An assessment of the durability of the performance of the OEIP over time was conducted on the sample with the highest current density obtained so far, which was PEDOT:PSS with a Nafion™-treated overoxidized region. Under the same applied voltage of 1 V, 50 cycles were repeatedly performed on the same sample to demonstrate how long the system could maintain its highest current density value (Figure 6b). After 50 repeating cycles it was observed that all current density values are approximately the same with a slight alteration of ±0.05 µA. This explains that the OEIP device developed here has a potential for long-term reproducibility and stable performance.

## 4. Conclusions

A flexible bioelectronic ion pump based on electrically-conductive polymers was fabricated using a line-pattering method and an enhancement of their electrical and ionic properties were performed. The availability and simplicity of this method makes it quite applicable and timesaving. Due to several surface modification techniques, the drop-casted conjugated polymer solution was better held on the surface, resulting in higher current values and ionic transfer. The electrical conductivity of PEDOT:PSS at a certain region was better tailored, optimizing the performance of the device. Unlike the chemical modification, electrochemical overoxidation, which better controlled the oxidation, and did not damage the surface, was performed on the PEDOT:PSS middle region. According to our best knowledge the ionic conductivity of the overoxidized PEDOT:PSS had been measured for the first time within this study. Additionally, the influence of the ionic conductivities of the electrochemically-oxidized region on the OEIP performances was tested. It was demonstrated that the influence of ionic conductivity of the electrochemically-overoxidized region on the OEIP performances were higher than that of the electrical conductivities of the electrodes. It was the first time with this study that the effects of ionic and electrical conductivities of the overoxidized region and electrodes, respectively, had been investigated. This promising progress in the field of organic electronics will lead to upcoming advancements in this technological field.

## Figures and Tables

**Table 1:** Surface roughness and contact angle values for pristine, overoxidized, and EG-treated PEDOT:PSS. A significant change in surface roughness and contact angle is observed between electrochemically-overoxidized and pristine PEDOT:PSS values, which is due to the change in surface energy.

Material	Contact Angle (Degree)	Surface Roughness
Overoxidized PEDOT:PSS	9.10	43.5
Pristine PEDOT:PSS	56.5	5.34
Ethylene Glycol Treated PEDOT:PSS	68.4	3.73

**Table 2:** Change in conductivity values for pristine, overoxidized, and EG-treated PEDOT:PSS. An increase in conductivity is observed after EG treatment, which led to rise in the amount of transported ions during potensiostatic measurements.

Material	Conductivity (S/cm)
Overoxidized PEDOT:PPS	0.063
Pristine PEDOT:PPS	0.38
Ethylene Glycol Treated PEDOT:PPS	8.0
Hydrochloric Acid Treated PEDOT:PPS (PH = 1.5)	34
Hydrochloric Acid Treated PEDOT:PPS (PH = 0.9)	54

**Table 3:** Enhancement in proton conductivity of the electrochemically-overoxidized region of OEIP. The enhancement of proton conductivity with Nafion™ largely increases the OEIP performance.

Material	Proton Conductivity (S/cm)
Nafion™ deposited electrochemically-overoxidized region	0.13
Pristine electrochemically-overoxidized region	0.0035
